# *ZmNF-YB16* Overexpression Improves Drought Resistance and Yield by Enhancing Photosynthesis and the Antioxidant Capacity of Maize Plants

**DOI:** 10.3389/fpls.2018.00709

**Published:** 2018-05-29

**Authors:** Baomei Wang, Zhaoxia Li, Qijun Ran, Peng Li, Zhenghua Peng, Juren Zhang

**Affiliations:** School of Life Sciences, Shandong University, Jinan, China

**Keywords:** maize, transgene, *ZmNF-YB16*, drought resistance, kernel yield, photosynthesis, antioxidant

## Abstract

ZmNF-YB16 is a basic NF-YB superfamily member and a member of a transcription factor complex composed of NF-YA, NF-YB, and NF-YC in maize. *ZmNF-YB16* was transformed into the inbred maize line B104 to produce homozygous overexpression lines. *ZmNF-YB16* overexpression improves dehydration and drought stress resistance in maize plants during vegetative and reproductive stages by maintaining higher photosynthesis and increases the maize grain yield under normal and drought stress conditions. Based on the examination of differentially expressed genes between the wild-type (WT) and transgenic lines by quantitative real time PCR (qRT-PCR), *ZmNF-YB16* overexpression increased the expression of genes encoding antioxidant enzymes, the antioxidant synthase, and molecular chaperones associated with the endoplasmic reticulum (ER) stress response, and improved protection mechanism for photosynthesis system II. Plants that overexpression *ZmNF-YB16* showed a higher rate of photosynthesis and antioxidant enzyme activity, better membrane stability and lower electrolyte leakage under control and drought stress conditions. These results suggested that *ZmNF-YB16* played an important role in drought resistance in maize by regulating the expression of a number of genes involved in photosynthesis, the cellular antioxidant capacity and the ER stress response.

## Introduction

Exposure to environmental stresses, such as drought, causes adverse effects on the growth and production of crops (Nakashima and Yamaguchi-Shinozaki, 2013). Maize is one of the most important foods and feed crops and is susceptible to drought during its growth and development, especially at the flowering stage (Shaw, 1977). Due to the increasing world population and a global water source shortage, breeding drought-tolerant maize and improving crop yields using biotechnological methods have great strategic significance (Que et al., 2014).

Nuclear factor Y (NF-Y), which is also known as CBF (CCAAT-binding factor) or HAP (heme activator protein), is a complex composed of three subunits [NF-YA (CBF-B or HAP2), NF-YB (CBF-A or HAP3) and NF-YC (CBF-C or HAP5)] (Sinha et al., 1995; Nardini et al., 2013). The NF-Y complex has been extensively studied in animal systems, and the highly conserved domain played an important role in the interaction between the NF-Y transcription factor protein and DNA (Mantovani, 1999). Each subunit is encoded by one gene in mammals and yeast. NF-YA makes sequence-specific contact with the CCAAT box (Xing et al., 1993). NF-YB and NF-YC, which are similar to histones H2A and H2B, respectively, form a tight dimer (Frontini et al., 2004) and then interact with NF-YA to form a heterologous trimeric complex (Romier et al., 2003). The NF-Y transcription complex interacts with other regulatory factors to activate or inhibit the expression of downstream genes (Wright et al., 1995; Coustry et al., 1998; Benatti et al., 2008). Unlike yeast and mammals, plants have numerous NF-Y subunit genes. For instance, 10 genes encode NF-YA, 13 genes encode NF-YB, and 13 genes encode NF-YC in *Arabidopsis* (Siefers et al., 2009). Currently, individual NF-Y subunits are known to play important roles in plant embryogenesis (Lee et al., 2003; Braybrook and Harada, 2008; Yamamoto et al., 2009; Mu et al., 2013), flowering time regulation (Kumimoto et al., 2008, 2010; Cao et al., 2014; Hwang et al., 2016), and abiotic stress resistance (Li et al., 2013; Sato et al., 2014; Siriwardana et al., 2014; Ma et al., 2015). Although maize (*Zea mays* L.) has even more NF-Y subunits, few studies have investigated the roles of these subunits to date. Some experimental evidence has shown that microRNA-169 and its target NF-Y family genes play an important role in maize under stress conditions (Luan et al., 2014, 2015). The high numbers of NF-Y subunits indicate the functional diversity of this family in plants. NF-Y is placed at the center of many developmental stress-responsive processes in the plant lineage (Petroni et al., 2012).

Based on comparative transcriptome data from inbred lines with different tolerances to drought stress, GRMZM2G384528 (Probe ID: Zm.13055.2.S1_at) was more highly expressed in the drought-resistant line H21 than in the drought-sensitive line Y478 after drought treatment (Zheng et al., 2010), which implied that GRMZM2G384528 might play important roles in the drought stress response of maize. GRMZM2G384528 was designated *ZmNF-YB16* according to the nomenclature of Zhang et al. (2016). Overexpression of *ZmNF-YB2*, which was also known as *ZmNF-YB13* (Zhang et al., 2016), conferred drought tolerance and improved corn yield under water-limited conditions (Nelson et al., 2007). Because of the functional diversity of this family in plants, we carried out a series of studies on *ZmNF-YB16* function, especially in the drought stress response. In this study, we cloned *ZmNF-YB16* and transformed it into the inbred maize line B104, and we searched the altered metabolic pathways in the *ZmNF-YB16* overexpression plants. Our investigation of *ZmNF-YB16* function indicated that photosynthesis was improved in transgenic plants, and the antioxidant capacity of the cells was enhanced by the generation of more antioxidant enzymes and compounds. Additionally, the expression of molecular chaperones increased, which was related to the misfolded protein response and the protection of the stability of photosynthesis system II. These results showed that *ZmNF-YB16* played an important positive role in drought resistance and improved kernel yields of maize.

## Experimental Procedures

### Plant Materials

The plant materials used in this study were the maize (*Z. mays* L.) drought sensitive inbred lines B104. Average transformation frequencies of B014 inbred was achieved 6.4% (Frame et al., 2005), therefore *Agrobacterium*-mediated transformation of B104 maize shoot tips (Li et al., 2008) was used to generate the transgenic plants. We used *Agrobacterium tumefaciens* strain LBA4404, which contained a mini-Ti pDAB101851-Prd29B::*ZmNF-YB16*-Pactin1::*bar*. The T-DNA region contained the coding sequence of *ZmNF-YB16* gene driven by the *Atrd29B* promoter from *Arabidopsis* and the *bar* gene from *Streptomyces hygroscopicus* driven by the *OsActin1* promoter. At the 3-leaf stage, the transformed plants were screened with the Liberty^®^ 280 SL herbicide [Willowood, United States, containing 24.5% (v/v) glufosinate-ammonium] at a 0.25% (v/v) final concentration. After 10 days, the surviving plants (T0) were transplanted to the field and self-pollinated to produce the T1 generation. Seeds from the T1 generation were sown and self-pollinated to produce the T2 generation. Herbicide resistance, PCR assay and southern blotting were used to select the positive plants in each generation. All of the transgenic lines used in this experiment were T3 generation plants. Three independent transgenic lines L59, L92, and L187, which had single copy insertion and higher expression levels were used for scientific research.

### PCR, Southern Blotting Analysis of the Transgenic Plants

The cetyltrimethylammonium bromide (CTAB) method was used to extract genomic DNA from the maize leaves. To ensure positive plants, specific primers matched with the bar gene were designed.

DNA from the positive plants was digested with *Hin*dIII and *Nde*I. *Nde*I was used to reduce the DNA fragment length of genome for no cutting site existed in the T-DNA region. Southern blotting was performed using a digoxigenin (DIG)-labeled Prd29B-ZmNF-YB16 specific probe as described in the DIG system manual (Roche, Inc., Basel, Switzerland).

### qRT-PCR Analysis of the Transgenic Plants

The TRIzol reagent [Sangon Biotech (Shanghai)Co., Ltd., China] was used to extract total RNA. DNase-treated RNA (500 ng) was used for cDNA synthesis with a RT reagent kit (Takara, Kyoto, Japan) according to the manufacturer’s protocol. qRT-PCR was performed on an ABI7500 qRT-PCR System with the SYBR^®^ RT-PCR Kit (Takara, Dalian, China). The maize actin1 gene was used as an internal control. *ZmNF-YB16* gene specific primer sequences were designed for qRT-PCR (**Supplementary Table S1**). The running procedure was 95°C 5 min for pre-degeneration, 95°C 15 s for degeneration, 54–62°C 15 s for annealing, 72°C 37 s for extension. 40 cycles were used in qRT-PCR. We used the mixed liquor without template as negative control. Relative gene expression levels were calculated using the Delta-Delta-*C*t method (Livak and Schmittgen, 2001). Three repeats were performed for the qRT-PCR analysis.

Gene expression pattern analysis was performed by using qRT-PCR. Samples as leaf, leaf tip, leaf base, root, kernel at VE, V1, V7 stage (referenced by MaizeGDB) were collected for *ZmNF-YB16* expression pattern analysis. To verify the RNA sequencing results in different growth stages, leaves of the WT and transgenic plants at the V3 stage were harvested for qRT-PCR. The primers are shown in **Supplementary Table S1**. Each experiment had three biological replicates.

### Osmotic Treatment During Seedling Growth

For osmotic treatment during seedling stage, nutrient solution with 14% (w/v) PEG_6000_ was used. Briefly, seeds from the transgenic and WT plants were surface sterilized with 0.1% (w/v) mercuric chloride for 5 min, washed five times with sterile water, and then placed in petri dishes (diameter: 12.5 cm) in the dark. After the seeds germinated, they were transferred to a plate (20 cm × 16 cm × 7 cm) and grown at a photon flux density of 250 μmol/m^2^/s (14 h/10 h, light/dark) at 32°C/25°C (day/night) in a green- house until the plants reached the 2- leaf stage. Then half of them were transformed into nutrient solution with 14% (w/v) PEG_6000_, another half were in the normal solution as control (Li et al., 2011). Five plants for each line were collected treated with 14% PEG_6000_ for 6 days for morphology analysis. For salt stress, nutrient solution with 100 mM NaCl was used and samples were collected for real-time RT-PCR analysis. Each experiment had three biologic repeats.

### Osmotic Stress Treatment of Plants

Seeds from the transgenic and WT plants were planted in plastic containers (60 cm × 40 cm × 14 cm) at 32°C/25°C (day/night) at a photon flux density of 250 μmol/m^2^/s (14 h/10 h, light/dark) green house with rich water until the seedlings grew to the four-leaf stage. There were four containers, and each container had 12 WT and 36 transgenic plants. Two of them contained a mixture of sand and vermiculite (Named S1, S2), and another two contained maize nutrient solution (Named N1, N2). Then, S1 were kept with 45–50% relative soil moisture (RSM) for 10 days (when RSM was reduced 45–50%, 1 day was recorded) to impose moderate drought stress (Avramova et al., 2015), and S2 were kept with 85–90% RSM as control conditions. RSM was measured once a day using a soil profile moisture meter (Tuopu TZS-P4, Zhejiang, China). Plants at 0 and 10 days were used to measure the relative water content (RWC). When sampled, the roots were rinsed carefully to wash off the mixture, 6 WT and 18 transgenic plants for phenotypic observation and other plants for biomass and root/shoot ratio determination. Straighten the plant, and we defined the plant height from stem node to the leaf top and the root length from the stem node to root bottom. N1 and N2 were irrigated with maize nutrient solution until V3 stage. Then N2 was irrigated with maize nutrient solution supplemented with 14% (w/v) PEG_6000_ for dehydration treatment. 0.1 g materials were harvested at 0, 24, 72, rehydration 12, 36 h for gene expression analyze and 0.2 g materials were harvested for enzyme activity determination. Each experiment had three biologic repeats.

### Drought Stress Treatment of Plants

The drought stress experiments at pre-flowering stage were performed under natural conditions during the maize growing season at Shandong University in Jinan, China. Seeds of WT and three T3 transgenic lines were sown into flower pots (height: 40 cm; diameter: 28 cm) fitted with homogenous soil in May with 1 plant sown per plot; the plants were watered sufficiently every day. At the 10-leaf stage, uniform and healthy plants were selected and divided into two groups. One group was served as non-stress control. Another group was subjected to drought stress treatment for 8 days by maintaining the soil water content (SWC) at approximately 15% (When SWC was reduced 15%, 1 day was recorded), and then this group was exposed to rehydration treatment. Physiological parameters including the photosynthetic parameters, photosystem II (PSII) activity, ion leakage, solute potential, and soluble sugar and proline contents were measured on 0, 1, 3, 5, and 8 days during the period of drought stress treatment and 4 days after rehydration. Materials at 0 and 3 days were used to measure the chlorophyll content. Samples tested were fully expanded leaves of similar sizes, ages and positions on the plants. Ten plants were planted for each line. Each experiment had three biologic repetitions.

A field experiment was performed in the Lvjia experiment field in Jinan (117°29′E, 36°54′N) at 2013 and 2014. The plots were arranged in a randomized complete block design with three replicates. Forty seedlings of each T3 transgenic and WT line were sown in a double-row plot in May (a population density of approximately 66700 plants/ha). The plot was 2.5 m in length, with a width of 1.2 m and an interval of 0.25 m between plants (Liu et al., 2013). Plants at the 10-leaf stage were subjected to drought stress for 6 weeks, with soli water content (SWC) maintained at 50–55% at a depth of 40 cm during stress. The plant height and anthesis-silking interval (ASI) of the maize plants were determined at the pollination stage. After harvesting, the ears were dried naturally to a constant weight, and the ear length, ear wide, hundred-grain dry weight, dry weight of per ear were recorded.

### Leaf/Shoot Physiological Measurement

The leaf disks from WT and transgenic plants were excised, and fresh weights (FWs) were recorded immediately. The disks were then soaked in deionized water overnight at 4°C and weighed (rehydrated weight, RW). And then the leaf disks dry weights (DWs) were obtained after drying in an oven at 80°C. The RWC was calculated as RWC (%) = (FW - DW)/(RW - DW) × 100%.

The net photosynthetic CO_2_ assimilation rate, stomatal conductance were measured by a portable infrared gas analyser-based photosynthesis system (LI-6400; LI-CoR Inc., Lincoln, NE, United States). The chlorophyll fluorescence was measured by using a pulse-modulation chlorophyll fluorometer (Mini-Pam; Walz, Effeltrich, Germany) at room temperature. The total soluble sugars in the maize leaves were extracted from 100 mg of material by immersing twice in boiling water for 30 min and measured with an anthrone reagent using glucose as the standard (Yemm and Willis, 1954). The solute potential (ψs) was measured with a cryoscopic osmometer (Model 210; Fiske, Norwood, MA, United States) and calculated with the formula: ψs = -moles of solute (RK), where *R* = 0.008314 and *K* = 298°C (Gaxiola et al., 2001). The electrolyte leakage in the leaf cells was measured with a conductivity meter (Kangyi DDS-320, Shanghai, China). The MDA content was determined using the protocol described by Quan et al. (2004). Proline was detected as described previously by Bates et al. (1973). Approximately 0.2 g of leaves was excised to measure chlorophyll content. Ca = 13.70^∗^OD663-5.76^∗^OD645; Cb = 25.96^∗^OD645-7.60^∗^OD663. The content of chlorophyll a = Ca^∗^0.025/0.2; Content of chlorophyll b = Cb^∗^0.025/0.2.

### The Measurement of GPX, GST, POD, and APX

0.2 g materials were used to measure the anti-oxidase enzyme activity. Measurement of glutathione peroxidase (GPX) activity was measured per the protocol of Alla et al. (2008). The glutathione *S*-transferase (GST) activity was measured as described by Liao et al. (2000). The total peroxidase (POD) activity was determined spectrophotometrically by measuring the oxidation of *o*-dianisidine (3, 3-dimethoxybenzidine) at 460 nm (Ranieri et al., 2000). The total ascorbate peroxidase (APX) activity was measured spectrophotometrically (Jimenez et al., 1997).

### Sequence Analysis

Multiple sequence alignments were generated using the DNAMAN 6.0.3.99 software (Lynnon Biosoft, Pointe-Claire, Quebec, Canada), and the phylogenetic tree was constructed using the MEGA (version 4.0) software (Kumar et al., 2008) and edited by Figtree (version 1.4.3). The *Arabidopsis*, maize and rice NF-YB family sequences from the PlantTFDB database (Jin et al., 2014).

### Statistical Analysis

The data were presented as the mean ± standard deviation (SD). Statistical analyses were performed using SigmaPlot 10.0 software (Systat Software Inc., Chicago, IL, United States). Comparisons between the transgenic and WT plants in **Table 1** were made using Duncan’s multiple-range tests with one-way ANOVAs in SPSS (version 22.0.0.0). Each experiment was performed on three independent biological replicates.

**Table 1:** Differences of the agronomic traits from maize plants grown under control conditions or suffered from drought stress in the fields.

Year	Materials	Conditions	Plant height (cm)	ASI (day)	Ear length (cm)	Ear wide (cm)	Grain weight/ear (g)	Hundred-grain dry weight (g)	Yield (kg/plot)
									
2013	WT	Well-watered	127.67 ± 2.05^cd^	2.93 ± 0.23^a^	12.53 ± 0.21^c^	3.46 ± 0.05^c^	56.11 ± 1.01^c^	21.7 ± 0.62^d^	2.26 ± 0.08^c^
		Drought	107.67 ± 2.62^a^	7.52 ± 0.25^c^	7.50 ± 0.15^a^	3.10 ± 0.05^a^	32.78 ± 1.34^a^	19.2 ± 0.22^a^	1.31 ± 0.03^a^
	L59	Well-watered	130.00 ± 2.16^d^	2.77 ± 0.15^a^	13.10 ± 0.35^c^	3.51 ± 0.11^d^	62.23 ± 1.07^d^	22.1 ± 0.51^e^	2.49 ± 0.04^d^
		Drought	118.67 ± 2.24^b^	5.03 ± 0.27^b^	10.33 ± 0.06^b^	3.41 ± 0.09^b^	54.28 ± 0.95^b^	20.5 ± 0.31b^c^	2.19 ± 0.11^b^
	L92	Well-watered	130.67 ± 4.49^d^	2.74 ± 0.10^a^	13.23 ± 0.22^c^	3.53 ± 0.06^d^	67.22 ± 1.07^e^	22.3 ± 0.26^e^	2.62 ± 0.08^e^
		Drought	122.62 ± 3.09^bc^	5.01 ± 0.10^b^	10.44 ± 0.12^b^	3.42 ± 0.13^bc^	55.77 ± 0.69^b^	20.7 ± 0.24^c^	2.21 ± 0.06^b^
	L187	Well-watered	130.77 ± 3.39^d^	2.67 ± 0.17^a^	13.33 ± 0.25^c^	3.10 ± 0.05^d^	66.11 ± 1.07^e^	22.9 ± 0.26^e^	2.61 ± 0.05^e^
		Drought	125.24 ± 2.05^c^	4.93 ± 0.29^b^	10.67 ± 0.34^b^	3.41 ± 0.04^b^	54.33 ± 1.52^b^	22.1 ± 0.42^e^	2.11 ± 0.04^b^
2014	WT	Well-watered	129.33 ± 3.60^c^	2.93 ± 0.21^a^	12.76 ± 0.17^d^	3.47 ± 0.05^c^	54.67 ± 0.94^c^	20.8 ± 0.45^c^	2.15 ± 0.04^c^
		Drought	108.67 ± 3.51^a^	7.36 ± 0.09^d^	7.40 ± 0.16^a^	3.10 ± 0.14^a^	32.67 ± 2.49^a^	18.9 ± 0.41^a^	1.36 ± 0.07^a^
	L59	Well-watered	132.00 ± 2.01^c^	2.80 ± 0.35^a^	13.12 ± 0.89^d^	3.50 ± 0.10^c^	60.00 ± 3.32^d^	21.6 ± 0.14^d^	2.35 ± 0.05^d^
		Drought	120.63 ± 3.46^b^	5.13 ± 0.28^c^	9.83 ± 0.60^b^	3.40 ± 0.08^b^	50.25 ± 2.67^b^	20.1 ± 0.61^b^	2.05 ± 0.07^b^
	L92	Well-watered	131.62 ± 3.10^c^	2.77 ± 0.17^a^	13.27 ± 0.41^d^	3.56 ± 0.14c	62.33 ± 4.01^d^	21.4 ± 0.52^d^	2.42 ± 0.08^d^
		Drought	119.87 ± 3.21^b^	4.86 ± 0.31^b^	9.80 ± 0.59^b^	3.43 ± 0.12^b^	52.57 ± 2.71^b^	20.3 ± 0.49^b^	2.09 ± 0.09^b^
	L187	Well-watered	131.07 ± 2.64^c^	2.73 ± 0.23^a^	13.31 ± 0.39^d^	3.50 ± 0.13^c^	61.33 ± 4.14^d^	22.3 ± 0.41^e^	2.42 ± 0.08^d^
		Drought	120.33 ± 3.51^b^	4.90 ± 0.18^bc^	10.13 ± 0.14^c^	3.36 ± 0.09^b^	51.03 ± 3.45^b^	20.4 ± 0.34^b^	2.10 ± 0.06^b^


**Values are mean ± SD and labeled with different letter are significant difference at P < 0.05 by Duncan’s test. Significance of difference analysis was tested within the same year. Each plot was arranged in a random complete block design with three replications.**

## Results

### Phylogenetic Relationship and Expression Pattern of *ZmNF-YB16*

**FIGURE 1:**
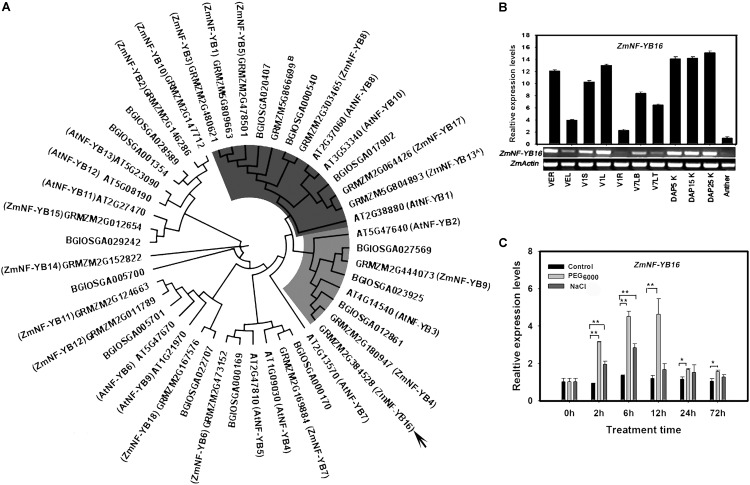
Phylogenetic relationship and expression pattern of *ZmNF-YB16*. **(A)** Phylogenetic relationships between maize, *Arabidopsis* and rice NF-YB family members. The *Arabidopsis*, maize and rice NF-YB family sequences from the PlantTFDB database (Jin et al., 2014). **(B)** Expression pattern of *ZmNF-YB16* in maize. R, root; S, stem; L, leaf; LT, leaf tip; LB, leaf base; K, kernel. The VE, V1, V7 stage was referenced by MaizeGDB. The expression levels were normalized to *ZmActin1*, and the level of *ZmNF-YB16* transcript in the anther was set as 1.0. **(C)** Expression of *ZmNF-YB16* in response to stress in maize. Plants in 3-leaf stage were used to assay the accumulation of *ZmNF-YB16* transcripts in response to 14% (w/v) PEG_6000_ and 100 mM NaCL solution. Superscript A: ZmNF-YB13 was named as ZmNF-YB2 by Nelson et al. (2007). Superscript B: GRMZM5G866699 was not identified by Zhang et al. (2016). Each experiment had three biologic repeats. The asterisks indicate a significant difference at ^∗^0.01 < *P* ≤ 0.05, ^∗∗^*P* ≤ 0.01.

The length of *ZmNF-YB16* coding sequence is 639 bp, which encodes a 212 amino acid protein with a predicted molecular mass of 23.3 kDa and an isoelectric point of 6.68. A phylogenetic tree was constructed using all NF-YB family members from rice, *Arabidopsis* and maize (**Figure 1A**). *ZmNF-YB16* and *ZmNF-YB13* [named as *ZmNF-YB2* by Nelson et al. (2007)] belonged to different subfamilies. And *AtNF-YB3* and *BGIOSGA012861* are the homologous genes of *ZmNF-YB16 in Arabidopsis* and Rice. A basic helix-loop-helix motif composed of four helices and three loops in the ZmNF-YB16 secondary structure was conserved in the NF-YB family members (**Supplementary Figure S1A**). The kernel at DAP 25 had the highest expression level, whereas expression was lowest in the anther (**Figure 1B**). To evaluate whether *ZmNF-YB16* expression was induced by osmotic stress, maize plants at 3-leaf stage were treated with 14% (w/v) PEG_6000_. When the plants were maintained under PEG_6000_ stress conditions for 2 h, *ZmNF-YB16* expression was induced obviously, and reached the peak at 12 h. Compared with dehydration, *ZmNF-YB16* was slightly affected by 100 mM NaCl stress (**Figure 1C**).

### Molecular Characterization of the Transgenic Maize Plants

The T-DNA region of the *ZmNF-YB16* vector plasmid (**Supplementary Figure S2A**) was transferred into the inbred maize line B104. The transgenic maize plants were confirmed using a PCR assay with modified *bar* gene-specific primers (**Supplementary Figure S2B**). To reduce the effect of *ZmNF-YB16* overexpression under control conditions, the *Atrd29B* promoter, which is an abiotic stress-inducible promoter in *Arabidopsis*, was selected to control *ZmNF-YB16* expression. qRT-PCR was used to estimate the relative expression of *ZmNF-YB16* in the transgenic and WT lines. Significant differences were detected between the WT and transgenic plants in control and drought conditions. The *ZmNF-YB16* transcript levels increased 1.83- to 2.49-fold in the transgenic plants and increased 1.2-fold in the WT plants after drought stress (**Supplementary Figure S2C**). PCR-positive plants in the T3 generation were confirmed using Southern blotting analysis. The DNA of three independent lines was digested by the restriction enzyme *Hin*dIII, which had one cutting site in the T-DNA region; due to random T-DNA insertion into the genome, fragments of different sizes were produced. An approximately 3.8-kb fragment represented the endogenous fragment (**Supplementary Figure S1B**), while the fragments with different sizes represented the transgenic fragments (**Supplementary Figure S2D**). *ZmNF-YB16* was stably integrated into the genome in the three independent transgenic lines.

### *ZmNF-YB16* Overexpression Improves Dehydration and Drought Stress Resistance in Maize Plants During Vegetative and Reproductive Stages

**FIGURE 2:**
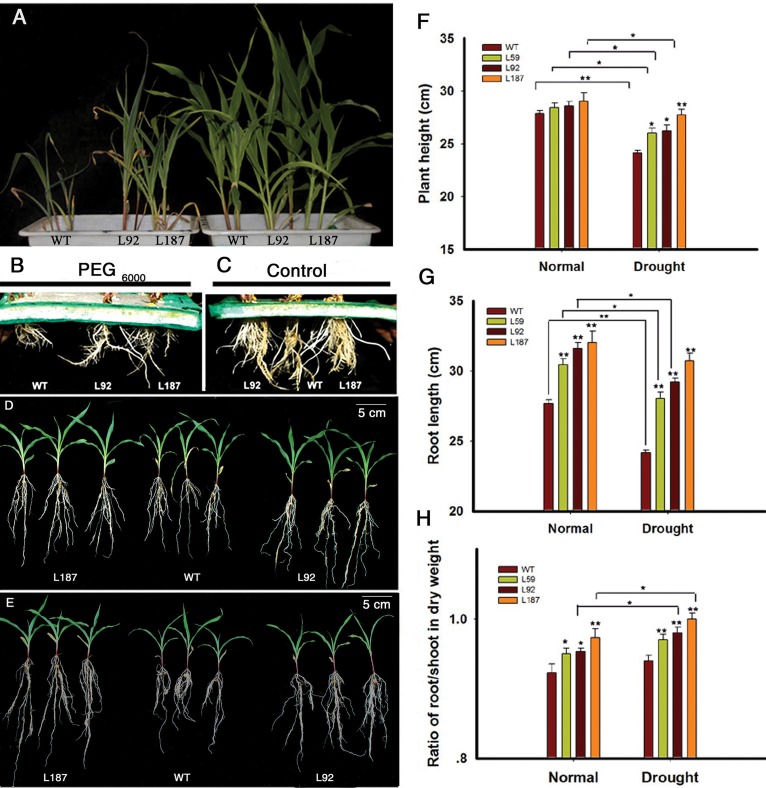
Characterizations of the *ZmNF-YB16* transgenic maize. **(A)** Difference of the aerial part between WT and *ZmNF-YB16* transgenic plants under control and osmotic stress conditions at 3-leaf stage. The left showed the plants treated with 14% PEG_6000_ for 6 days. The right showed the plants under control conditions. **(B)** Root system of different lines after osmosis stress. **(C)** Root system of different lines under control conditions. **(D)** Morphology of the plants from different lines under control conditions. **(E)** Morphology of the plants from different lines after drought treatment. **(F–H)** Plant height, root length and the ratio of root to shoot under control and drought conditions, respectively. Plants **(A–C)** were at 3-leaf stage. Plants **(D,E)** were at 5-leaf stage. Data are mean ± SD. Five biological repeats were used. Asterisks denote significant differences: ^∗^0.01 < *P* ≤ 0.05, ^∗∗^*P* ≤ 0.01.

At the 3-leaf stage, 14% (w/v) PEG_6000_ treatment was used to create a dehydration environment. The aerial parts of the transgenic lines were slightly larger than the aerial parts of the WT plants under control conditions. However, significant differences were apparent after treatment with 14% (w/v) PEG_6000_ for 6 days. The growth of the WT plants was slower, and their wilting was more severe than the transgenic lines (**Figure 2A**). Interestingly, the root system, especially the lengths of the seminal roots and the numbers of lateral roots, exhibited apparent differences under both control and osmotic stress conditions (**Figures 2B,C**).

At the 5-leaf stage, plants in soil with 80–85% RSM (**Figure 2D**) and 45–50% RSM (**Figure 2E**) had significant differences, and a root system phenotype similar to the phenotype in the 3-leaf stage was observed. The leaf RWC was measured. There was no difference between WT and transgenic plants in control conditions. When treated with drought for 10 days, transgenic plants kept 82–86% RWC, whereas RWC in WT decreased to 73% (**Supplementary Figure S3**). To evaluate these differences, we measured plant height, root length and the root/shoot ratio (**Figures 2F–H**). The root/shoot ratio increased after drought stress in all plants. The WT plants exhibited an increase in the root/shoot ratio from 0.92 to 0.94, whereas the transgenic line L187 exhibited an increase from 0.97 to 1.01.

**FIGURE 3:**
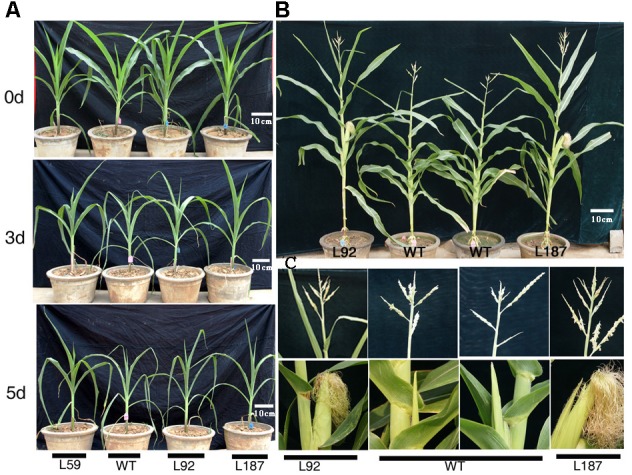
Phenotypic differences of *ZmNF-YB16* overexpression lines and WT in pre-flowing period. **(A)** The growth status of WT and transgenic plants before and after drought stress. 0, 3, and 5 days represent the drought treatment time. **(B)** The distinguishable differences of maize after drought stress between WT and transgenic plant (drought stress for 3 weeks beginning at the 10-leaf stage). **(C)** The enlarged parts of the ear and tassel of plants on **(B)** photograph. L92, L187: *ZmNF-YB16* overexpression lines; WT, B104 Wild type.

At the 10-leaf stage, no obvious phenotypic differences were observed between the transgenic lines and the WT plants under control conditions in our study. However, all of the plants gradually wilted and showed different degrees of damage as the duration of the drought stress increased. The transgenic lines grew better and were less withered than the WT plants. After exposure to drought stress for 8 days, delayed growth was observed in all lines. However, the drought stress had a reduced effect on the growth of the transgenic plants, especially on plant height and reproductive organ development (**Figure 3**). The number of tassel branches and the activity of pollen were much greater and the anthesis-silking interval (ASI) of the transgenic plants was shorter than those in WT plants (**Table 1**).

### *ZmNF-YB16* Overexpression Increases the Maize Grain Yield Under Normal and Drought Stress Conditions

**FIGURE 4:**
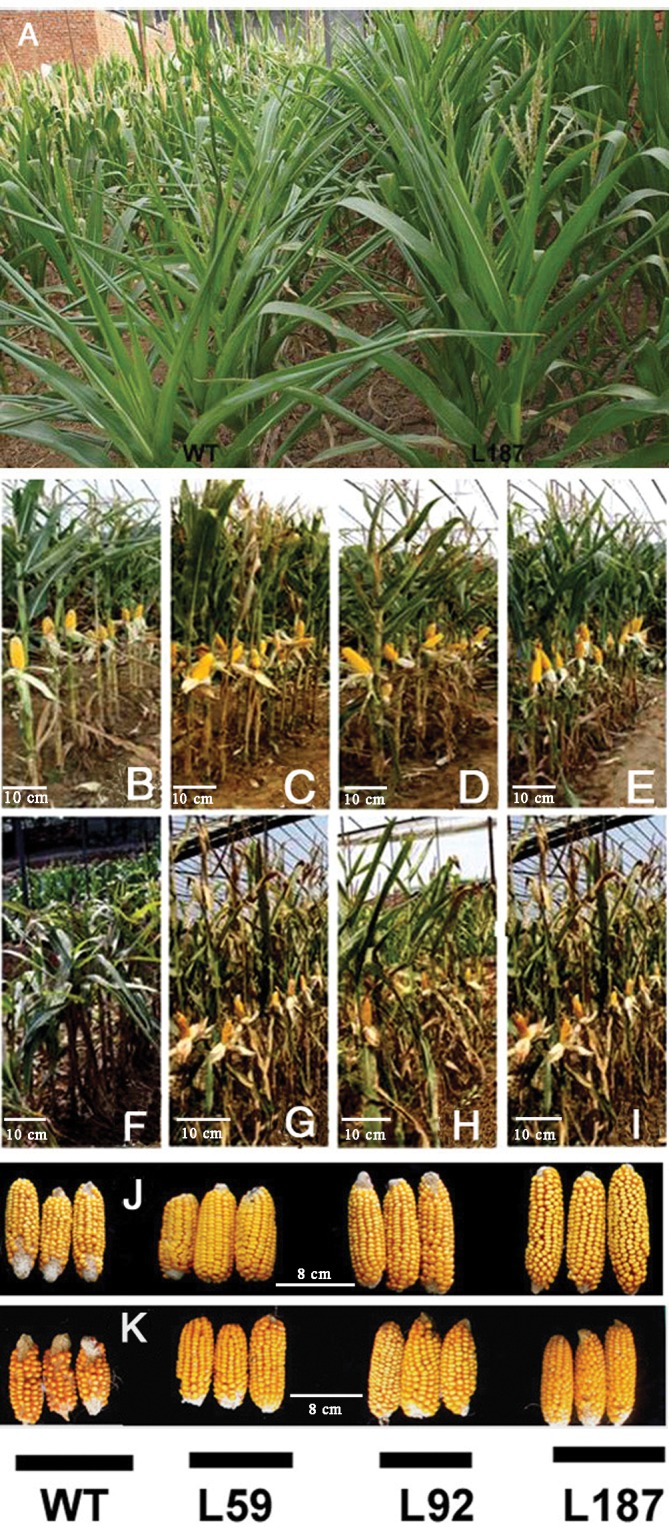
Phenotypes of overexpressing *ZmNF-YB16* lines in the fields. **(A)** The distinguishable differences of maize between WT and transgenic plants after drought treatment. **(B–E)** Plants grew under control conditions. **(F–I)** Plants suffered from drought stress for 6 weeks during pre-flowering phase. **(J)** Ears from plants under control conditions. **(K)** Ears from plants suffered from drought stress for 6 weeks. **(B,F)**: WT; **(C,G)**: L59; **(D,H)**: L92; **(E,I)**: L187. WT, non-transformed control; L59, L92, L187: *ZmNF-YB16* transgenic plants.

In the field studies, male and female organ development, were observed between the WT and transgenic plants under drought conditions (**Figure 4A**). Under control conditions, the growth and development of the plants exhibited no obvious differences between the transgenic and non-transgenic lines (**Figures 4B–E**). However, after drought stress for 6 weeks from the 10-leaf stage, significant differences were found between the lines. For instance, the WT plants were shorter and had aborted ears (**Figure 4F**). Although the height and ears of the transgenic plants were also affected by drought, their state was much better than that of the WT plants (**Figures 4G–I**). The agronomic traits of the WT and transgenic maize plants under both control and drought conditions were recorded and calculated in 2013 and 2014, including ear length, cob width, hundred-grain dry weight and grain dry weight per ear (**Table 1**). Pictures of the ears from both the WT and transgenic plants are shown in **Figure 4J** (control) and **Figure 4K** (drought). No obvious differences were found in cob width between the WT and transgenic plants. In contrast, the differences in the hundred-grain dry weight and the grain dry weight per ear were significant. The hundred-grain dry weight under control conditions was 21.7 g for the WT plants and 22.9 g for transgenic line L187. After drought stress for 6 weeks, the hundred-grain dry weight decreased to 19.2 g in WT plants and 22.1 g in transgenic line L187. Likewise, under control conditions, the block crop yield of the WT plants was 2.26 kg, but the block crop yields of the transgenic lines ranged from 2.49 to 2.61 kg, which represented 1.10–1.16-fold of the WT yield. After drought stress, the block crop yield of the WT plants was 1.31 kg, but the block crop yield of the transgenic lines ranged from 2.11 to 2.21 kg, which represented a 1.61–1.69-fold of the WT yield.

### *ZmNF-YB16* Is Involved in the Protection of Photosynthesis System II

**FIGURE 5:**
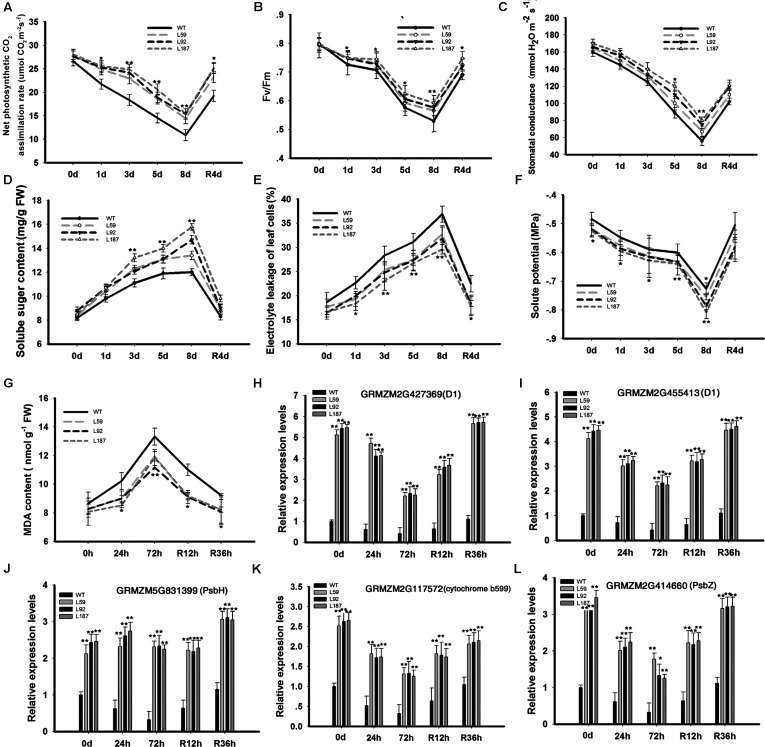
Changes of physiological characteristics and gene expression related to photosynthesis system and member stability in transgenic and WT plants. Plants at pre-flowering stage were used to analyze the physiological characteristics. 0, 1, and 8 days meant the drought treatment time and R4d meant the rehydration time. Leaves at V3 stage were used to analyze the gene expression levels. 0, 24, and 72 h represented the drought treatment time, and R12h and R36h represented the rehydration time. WT, wild type; L59, L92, L187: transgenic lines. **(A–G)** Physiological characteristics analysis; **(H–L)** Gene expression analysis. The asterisks indicate the significant difference between transgenic plants and WT, ^∗^0.01 < *P* < 0.05, ^∗∗^*P* < 0.01. Each experiment had three biologic repeats.

To determine why the plants with *ZmNF-YB16* overexpression had higher grain yields, net photosynthetic CO_2_ assimilation rates (**Figure 5A**), chlorophyll fluorescence (*F*_v_/*F*_m_) (**Figure 5B**), stomatal conductance (**Figure 5C**), and soluble sugar content (**Figure 5D**), were measured in the WT and transgenic plants under normal and drought conditions. No significant differences were found under normal conditions. However, the net photosynthetic CO_2_ assimilation rates of the transgenic plants were significantly higher than the rates of the WT plants under drought stress conditions. On the 8th day of stress, the stomatal conductance of the WT plants was reduced to 56.0 mmol H_2_O m^-2^s^-1^, the *F_v_*/*F_m_* of the WT plants decreased to 0.5, and the net photosynthetic CO_2_ assimilation rate of the WT plants declined to 9.8 μmol CO_2_ m^-2^s^-1^, while these parameters in the transgenic line L187 were 83.1 mmol H_2_O m^-2^s^-1^, 0.6 and 16.3 μmol CO_2_ m^-2^s^-1^, respectively. When the plants were rehydrated, these parameters appeared to show a greater increase in the transgenic plants than in the WT plants.

To detect solute accumulation in the leaf cells of the maize plants during drought stress, the leaf solute potential (Ψs) was determined in the plants (**Figure 5F**). No significant differences were found between the WT and transgenic plants before drought stress or on the 1st and 3rd days of stress. However, on the 8th day of drought stress, the differences in the Ψs parameters were significant. These differences were still present on the 4th day of rehydration.

Next, we investigated why the transgenic plants had better photosynthesis abilities than did the WT plants under drought stress conditions. We analyzed the gene expression levels in photosynthesis system (**Figures 5H,I**). Up-regulated genes in the transgenic plants compared to the WT plants under drought conditions included GRMZM5G831399 (encoding the photosystem II PsbH protein), GRMZM2G427369, GRMZM2G455413 (encoding the photosystem II P680 reaction center D1 protein), GRMZM2G414660 (encoding the photosystem II PsbZ protein), and GRMZM2G117572 (encoding the photosystem II cytochrome b599 subunit) (**Figures 5J–L**). PsbZ is the linker between light harvest complex II (LHCII) and the PSII core, and PsbH is important for PSII stabilization and assembly (Shi and Schroder, 2004). At the same time, chlorophyll a and b content were measured (**Supplementary Figure S4**). Chlorophyll a did not differ between WT and transgenic plants, but chlorophyll b was higher in transgenic plants than in WT in both control and drought conditions. In control conditions, there was no remarkable difference in the photosynthetic parameters, but the expressions of PSII subunit genes were much higher in transgenic plants.

### *ZmNF-YB16* Alleviates Drought-Induced Oxidative Stress by Improving the Antioxidant Capacity and Membrane Protection of the Cell

**FIGURE 6:**
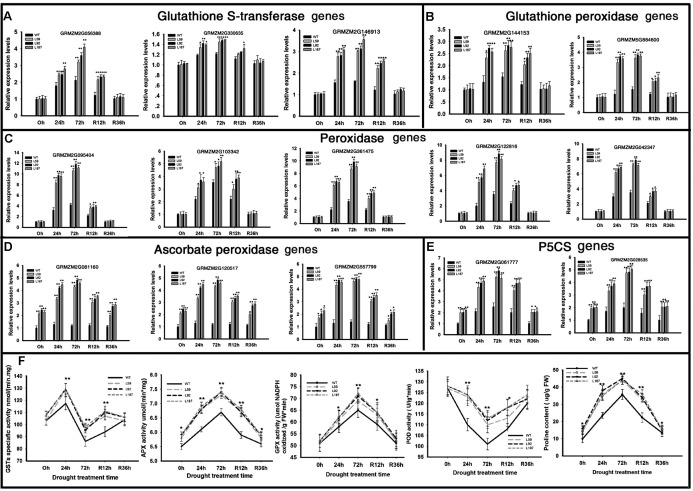
Transcript levels of genes and physiological characteristics involved in anti-oxidation in transgenic and WT plants. **(A–E)** Gene expression analysis; **(F)** physiological characteristics analysis. Leaves at V3 stage were used to determine the gene expression levels. 0, 24, and 72 h represented the drought treatment time. R12h and R36h represented the rehydration time. WT: wild type; L59, L92, L187: different transgenic lines. GST, glutathione *S*-transferase; APX, ascorbic acid hydrogen peroxide enzyme; GPX, glutathione peroxide; POD, peroxidase. The asterisks indicate a significant difference between the transgenic line and the WT at ^∗^0.01 < *P* ≤ 0.05, ^∗∗^*P* ≤ 0.01. Each experiment had three biologic repeats.

To determined antioxidant ability, POD, GST, APX, and GPX activity were measured (**Figure 6F**). After drought stress, the enzyme activity of overexpression lines also kept higher levels than WT plants. When rehydrated, the overexpression lines had faster recoverability. All of the results showed that *ZmNF-YB16* overexpression substantially increased the antioxidant capacity of the cell.

We analyzed the gene expression levels in oxidative stress response system (**Figures 6A–E**). Three GST genes were dramatically induced by *ZmNF-YB16* overexpression, especially the GST coding gene GRMZM2G056388, which was increased 4.21-fold compared to the level in the WT in drought conditions. Ten peroxidase-encoding genes were up-regulated in overexpression lines after drought stress. Two of these genes were annotated to glutathione peroxidase (**Figure 6B**). Three of these genes were annotated to the ascorbate peroxidase (**Figure 6D**). The higher expression levels of these genes in *ZmNF-YB16* overexpression lines may contribute to the better oxidative stress response system.

Proline content of the overexpression lines was slightly higher than that in the WT plants under normal conditions. Under drought stress, the transgenic plants had significantly higher proline content than did the WT plants, although the WT plants had higher proline content prior to the stress (**Figure 6F**). We analyzed the gene expression levels of 1-pyrroline-5-carboxylate synthetase (P5CS) genes. One delta P5CS gene (GRMZM2G061777) in the *ZmNF-YB16* overexpression lines exhibited approximately 2.97-fold higher expression under control conditions and 2.45-fold higher expression under drought conditions compared to their expression levels in the WT plants. Another P5CS2 gene, GRMZM2G028535, also had higher expression levels in the *ZmNF-YB16* overexpression plants. We also measured membrane damage in the WT and transgenic plants before and after drought stress using two crucial indices: electrolyte leakage (**Figure 5E**) and MDA content (**Figure 5G**). The electrolyte leakage (%) from leaf cells gradually increased in all plants as drought progressed. However, the damage to the transgenic plants was much lower than the damage to the WT plants. For instance, on the 8th day of drought stress, the electrolyte leakage (%) in the WT plants was 36.9%, which was much higher than the electrolyte leakage in the transgenic lines (29.6–33.7%). Significant differences were also observed on the 4th day of rehydration. Electrolyte leakage, MDA and proline content were all affected by drought, and *ZmNF-YB16* overexpression, and the transgenic plants had favorable levels under drought stress, showing that the transgenic plants had less cellular membrane injury and better cellular stability.

### Overexpression of *ZmNF-YB16* Promotes the Expression of Molecular Chaperones to Improve Drought Stress Resistance

**FIGURE 7:**
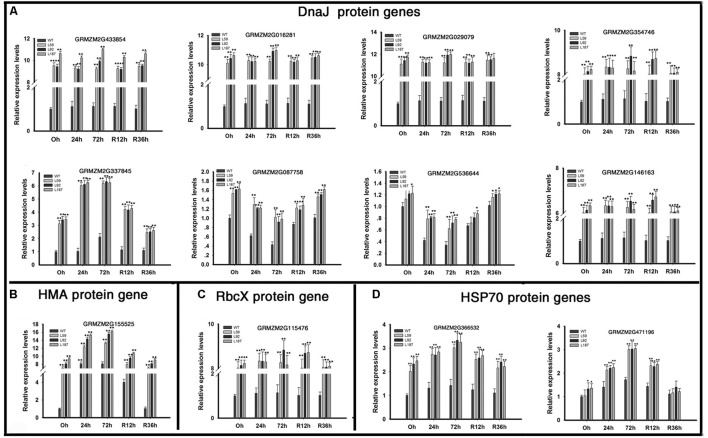
Transcript levels of molecular chaperone genes in transgenic and WT plants. Leaves at V3 stage were used to analyze the gene expression levels. 0, 24, and 72 h represented the drought treatment time. R12h and R36h represented the rehydration time. DnaJ protein: Heat shock protein 40. HAM, heavy metal transport/detoxification domain; HSP70, heat shock protein 70; RbcX, Chaperonin-like Rubisco X protein. WT, wild type; L59, L92, L187: different transgenic lines. The asterisks indicate a significant difference between the transgenic line and the WT at ^∗^0.01 < *P* ≤ 0.05, ^∗∗^*P* ≤ 0.01. Each experiment had three biologic repeats. **(A)** The expression levels of DnaJ protein genes. **(B)** The expression levels of HMA protein genes. **(C)** The expression levels of RbcX protein genes. **(D)** The rxpression levels of HSP70 protein genes.

Dramatic up-regulation of ER protein-processing genes, especially the DnaJ protein genes, were found in the overexpression lines (**Figure 7**). Four *AtJ3* homolog genes (GRMZM2G433854, GRMZM2G016281, GRMZM2G029079, GRMZM2G354746) showed large differences in expression between the WT and transgenic plants under normal conditions; these genes, were expressed at 10.84-, 11.24-, 11.41-, and 8.35- fold higher levels in the *ZmNF-YB16* overexpression lines than in the WT plants under drought conditions. One *AtJ2* homologs genes (GRMZM2G337845), one *AtDJC82* homologs genes (GRMZM2G087758), one *AtDJA7* homologs genes (GRMZM2G536644), and one *AtJ20* homologs gene (GRMZM2G146163) were also differentially expressed under normal and drought conditions. An HMA domain protein gene (GRMZM2G155525) and a chaperonin-like RbcX protein (GRMZM2G115476), which acted as a chaperone during the folding of Rubisco, also had higher expression levels in transgenic plants. Two HSP70 coding genes, GRMZM2G366532 and GRMZM2G471196, had higher expression levels in the overexpression lines under normal conditions. The different expression levels of these molecular chaperones in WT and transgenic plants may result the different drought resistance.

## Discussion

### *ZmNF-YB16* and *ZmNF-YB2/ZmNF-YB13* Belong to Different Clans of the NF-YB Subfamily

Nelson reported that transgenic maize plants with increased *ZmNF-YB2/ZmNF-YB13* expression showed drought tolerance under water-limited conditions based on the responses of a number of stress-related parameters, including chlorophyll content, stomatal conductance, leaf temperature, reduced wilting, and maintenance of photosynthesis (Nelson et al., 2007). In this study, *ZmNF-YB16* overexpression improved drought tolerance and contributed to a higher grain yield in maize in water-limited environment. *ZmNF-YB16* and *ZmNF-YB2/ZmNF-YB13* belong to different clans and is most closely related to *AtNF-YB3* (**Figure 1A**). Higher *ZmNF-YB16* expression has been reported in the primary roots, stem, shoot apical meristem (SAM), different parts of the leaf, the first internode, and the endosperm, whereas *ZmNF-YB2/ZmNF-YB13* exhibited higher expression mainly in the embryo, stem, SAM, immature tassel, and shoot tip (Zhang et al., 2016). When the plants were subjected to osmotic stress, the expression levels of both *ZmNF-YB16* and *ZmNF-YB2/ZmNF-YB13* were remarkably increased, but *ZmNF-YB2/ZmNF-YB13* showed a slower reaction. The results showed that both *ZmNF-YB16* and *ZmNF-YB2/ZmNF-YB13* were stress response genes with different native roles in response to the environment, but they showed obvious differences in organ expression pattern or in stress response rate.

### *ZmNF-YB16* Overexpression Maintain the Kernel Yield of Maize Under Drought Conditions by Enhancing Photosynthesis and Anti-oxidation Capacity

*Atrd29B* is induced within 3 h of dehydration in *Arabidopsis* (Yamaguchi-Shinozaki and Shinozaki, 1994). Under control conditions, *ZmNF-YB16* showed slightly higher expression in the transgenic plants in which the transgene was driven by Prd29B, which was consistent with other related studies using Prd29B in our laboratory. The maize transgenic lines showed higher yields than the WT plants under control and water-limited conditions in the fields (**Table 1**). This phenomenon provides a breeding strategy for new maize varieties with improved resistance to drought stress.

PSII is a multi-subunit protein pigment complex containing polypeptides that is located in the thylakoid membrane and is composed of approximately 20 subunits with a core pseudo-symmetric heterodimer containing two homologous proteins [D1 (PsbA) and D2 (PsbD)] as the reaction centre (Shi and Schroder, 2004). Cytochrome b-559 may function to protect photosystem II from photoinhibition (Thompson and Brudvig, 1988). PSII is very sensitive to changes in the environment, and the activity of PSII declines more rapidly than many other physiological activities under unfavorable or stressful environmental conditions (Figueroa et al., 2003). Transgenic expression of violaxanthin de-epoxidase (VDE), PsbS, and zeaxanthin epoxidase (ZEP) in combination in tobacco led to a more rapid recovery of the efficiency of photosynthetic CO_2_ assimilation in the shade (Kromdijk et al., 2016) and improved photosynthesis and crop productivity. The small chloroplast DnaJ proteins AtJ8, AtJ11, and AtJ20 participate in the optimization of CO_2_ fixation, stabilization of the PSII complexes and balance of electron transfer reactions (Chen et al., 2010). In our study, the genes coding PSII subunits and DnaJ proteins may have played important roles in photosynthesis protection in the *ZmNF-YB16* overexpression lines under stress conditions. Photosynthesis is a process consisting of many steps. We speculated that the regulation of PSII subunit genes at the transcriptional level may not be rate-limiting, or other steps limited the photosynthetic rate, stomatal conductance and *F*_v_/*F*_m_.

Oxidative stress is a major physiological mechanism underlying the trade-off between growth, reproduction, and defense. The ability of a plant to control oxidant levels is highly correlated with stress acclimation (Gill and Tuteja, 2010). Thylakoid ascorbate peroxidase (tAPX) is thought to be a key regulator of intracellular H_2_O_2_ levels (Duan et al., 2012). Overexpression of *tAPX* genes in tomatoes conferred tolerance to cold stress by maintaining higher reduced glutathione (GSH), chlorophyll content and APX activities compared to those in the WT plants (Duan et al., 2012). Catalases and APXs play important roles in the removal of hydrogen peroxide from the cell (Queval and Foyer, 2012). The mechanisms normally include the induction of genes encoding antioxidants, cell rescue and defense proteins, as well as signaling proteins, such as kinases, phosphatases, and transcription factors (Hung et al., 2005). A variety of genes encoding antioxidant enzymes and antioxidant compounds showed differential expression between the WT and overexpression lines under control and drought conditions.

An increased investment in defense mechanisms usually reduces growth capacity, leading to negative effects on growth and reproduction. However, defense responses are essential for survival. If the plant is repeatedly exposed to these stressors, as is common in nature, investments in defense not only guarantee survival but also can allow optimization of long-term reproductive fitness under stress conditions (Morales and Munne-Bosch, 2016). This finding is consistent with the higher yield of the *ZmNF-YB16* overexpression lines.

*ZmNF-YB16* is a potential Opaque2 (O2) direct target gene, and O2 is a transcription factor that plays important roles during maize endosperm development (Li et al., 2015). Therefore, ZmNF-YB16 may play important roles in endosperm development. With the improvement of photosynthesis and antioxidant properties *ZmNF-YB16* overexpression maintains the higher kernel yield of maize under drought conditions.

### *ZmNF-YB16* Regulates the Maize Stress Response

*AtNF-YB3* is the orthologous gene of *ZmNF-YB16* in *Arabidopsis*. The transcriptional complex consists of an AtNF-YA4/AtNF-YB3/AtNF-YC2 trimer and bZIP28, which plays an important role in the ER stress response (Liu and Howell, 2010). DPB3-1 (AtNF-YC10) can form a transcriptional complex with AtNF-YA2/AtNF-YB3; the identified trimer enhanced heat stress-inducible gene expression during the heat stress response in cooperation with DREB2A (Sato et al., 2014). Overexpression of *HAP3b* (*AtNF-YB2*) enhanced primary root elongation in *Arabidopsis* (Ballif et al., 2011). AtNF-YB2 and AtNF-YB3 play additive roles in the promotion of flowering by inducing long-day photoperiods in *Arabidopsis* (Kumimoto et al., 2008). These studies demonstrated the functional diversity of *AtNF-YB3*, especially in relation to stress response. Drought at the flowering stage often results in barrenness (Edmeades et al., 1999). In this study, plant growth status and reproductive organ development suffered less damage in the transgenic plants and physiological characteristics related to drought resistance were all improved in the transgenic plants compared to those in the WT plants.

Different antioxidant systems dominate in specific parts of the growth zone during drought stress (Avramova et al., 2015). The leaf meristems are better protected during the stress, particularly due to higher activities of the redox-regulating enzymes CAT, POX, APX, and GR, which results in less H_2_O_2_ production and allows improved growth under drought conditions (Avramova et al., 2017). APX is a key enzyme in the ASA (ascorbic acid)-GSH (glutathione) cycle that plays an important role in reactive oxygen species (ROS) scavenging. This enzyme mainly deoxygenates the H_2_O_2_ that accumulates in the cytoplasm and chloroplasts into H_2_O and O_2_ to avoid cell poisoning (Shigeoka et al., 2002; Ishikawa and Shigeoka, 2008). Drought stress invariably leads to oxidative stress in plant cells due to higher leakage of electrons to O_2_ during photosynthesis and respiratory processes, leading to greater generation of activated oxygen species (Stepien and Klobus, 2005). The increased expression of antioxidant enzyme genes may contribute to ROS scavenging. This process protects the cell from damage due to the oxidation of proteins, inactivation of enzymes, alterations in gene expression, and decomposition of membranes.

Pyrroline-5-carboxylate synthase (P5CS) is the key enzyme in the synthesis of proline and ornithine (Perez-Arellano et al., 2010). Proline accumulation results from an increase in P5CS transcription, an increase in P5CS activity, and/or the inhibition of proline degradation, which is reduced in plants under water stress conditions (Perez-Arellano et al., 2010). The higher proline content in transgenic plants under drought conditions may protect the plants from damage.

Abiotic stresses, such as heat, cold, freezing, drought, or oxidizing agents, usually cause protein dysfunction. Protein folding stability is undoubtedly one of the most challenging problems for an organism undergoing stress (Hammarstrom and Carlsson, 2000). Chaperones are responsible for protein folding, assembly, translocation and degradation in many cellular processes, and those such as Hsp70 has essential functions in preventing aggregation and in assisting in the refolding of non-native proteins under both normal and stress conditions (Hartl, 1996; Frydman, 2001). In our study, the genes encoding molecular chaperone made positive contributions to drought stress response in overexpression lines.

We concluded that *ZmNF-YB16* overexpression enhanced drought resistance in maize at the seedling, pre-flowering, and reproductive stages and maintained grain yield under drought conditions. Moreover, the most overrepresented activities in the *ZmNF-YB16* overexpression lines were related to the protection of antioxidant enzymes capacity, PSII and ER protein processing.

Generally, seeds produced in self-cross will suffer a setback, which is a decrease in seed quality. And that will eventually raise the overall changes in the seeds and result in reduced viability of seeds. Therefore, we are not sure whether the good traits will be maintained after multiple self-cross in transgenic plants. But there is no doubt that the application of *ZmNF-YB16* could improve yields under areas with sufficient and minimal water inputs in maize production.

## Author Contributions

JZ: experiments design. BW: experiments operation and writing - original draft. BW and QR: methodology. PL and ZP: software. BW and ZL: data curation. BW and JZ: writing - review and editing.

## Conflict of Interest Statement

The authors declare that the research was conducted in the absence of any commercial or financial relationships that could be construed as a potential conflict of interest.

## Supplementary Material

FIGURE S1 Multiple amino acid sequence alignment of *ZmNF-YB16* and homologous genes and T-DNA region sequences and Location of the endogenous gene on chromosome 7.

FIGURE S2 Schematic structure of the T-DNA region of plasmid pDAB101851- Prd29B::*ZmNF-YB16*.

FIGURE S3 Relative water content of WT and transgenic plants under normal and drought conditions.

FIGURE S4 Chlorophyll content of WT and transgenic plants under normal and drought conditions.

TABLE S1 List of primers used in this research.
