# Supplementary material for: ZmNF-YB16 Overexpression Improves Drought Resistance and Yield by Enhancing Photosynthesis and the Antioxidant Capacity of Maize Plants
Source: Front Plant Sci. 2018 May 29;9:709. doi: 10.3389/fpls.2018.00709 (PMC5986874; doi:10.3389/fpls.2018.00709)
Supplement: FIGURE S1 — Multiple amino acid sequence alignment of ZmNF-YB16 and homologous genes and T-DNA region sequences and Location of the endogenous gene on chromosome 7. [file Image_1.PDF]

ZmNF-YB16 overexpression improves drought resistance and yield by enhancing photosynthesis and the antioxidant capacity of maize plants

Baomei Wang, Zhaoxia Li, Qijun Ran, Peng Li, Zhenghua Peng, Juren Zhang*

***Corresponding author:**
Juren Zhang
jrzhang@sdu.edu.cn

Supplemental data 1

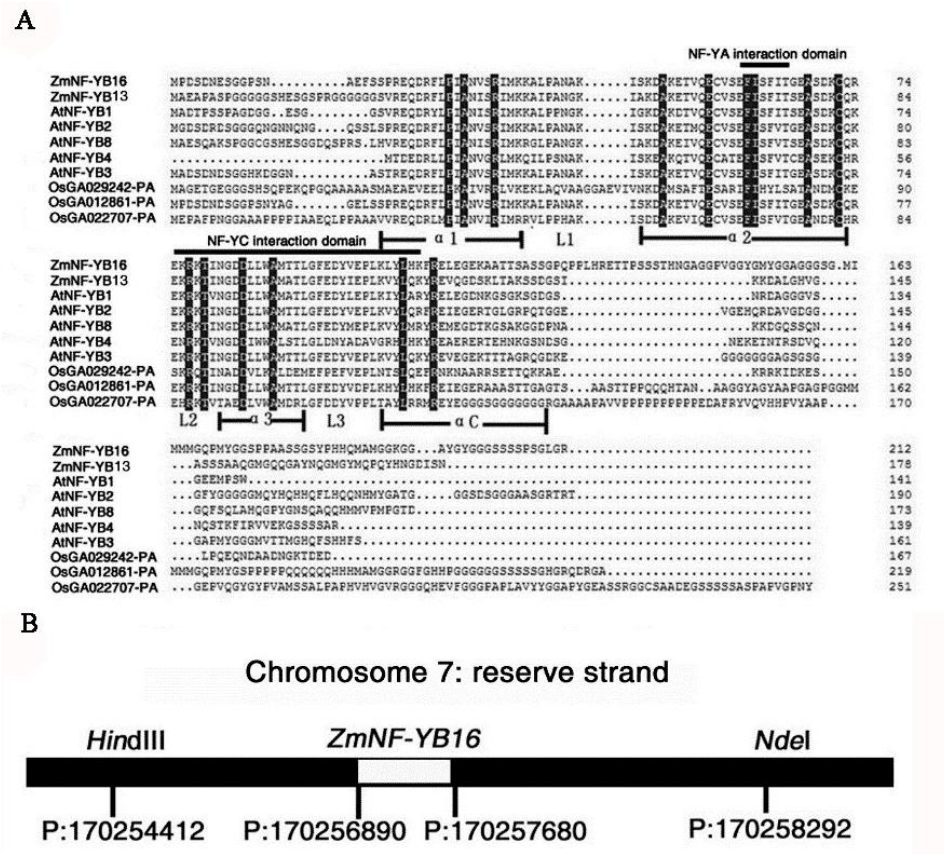

Figure S1 Multiple amino acid sequence alignment of ZmNF-YB16 and homologous genes and location of endogenous gene on chromosome 7

(A) Multiple amino acid sequence alignment of *ZmNF-YB16* and homologous genes in maize, rice, and Arabidopsis. Os: Oryza sativa; Zm: Zea may; At: *Arabidopsis thaliana*. Numbers in the right side correspond to actual amino acid numbers. Sequences in dark blue shadows mean the conversed amino acid in all the genes. The α1, α2, α3, αC represent the helix in secondary structure; L1, L2, L3 represent the β-strands in secondary structure. ZmNF-YB16: GRMZM2G384528; ZmNF-YB2:GRMZM5G804893; AtNF-YB1:AT2G38880; AtNF-YB2:AT5G47640; AtNF-YB3:AT4G14540; AtNF-YB4:AT1G09030; AtNF-YB8:AT2G37060. (B) The location of endogenous gene in chromosome 7. P: position. The location was referenced by GRAMENE (Tello-Ruiz et al., 2016).

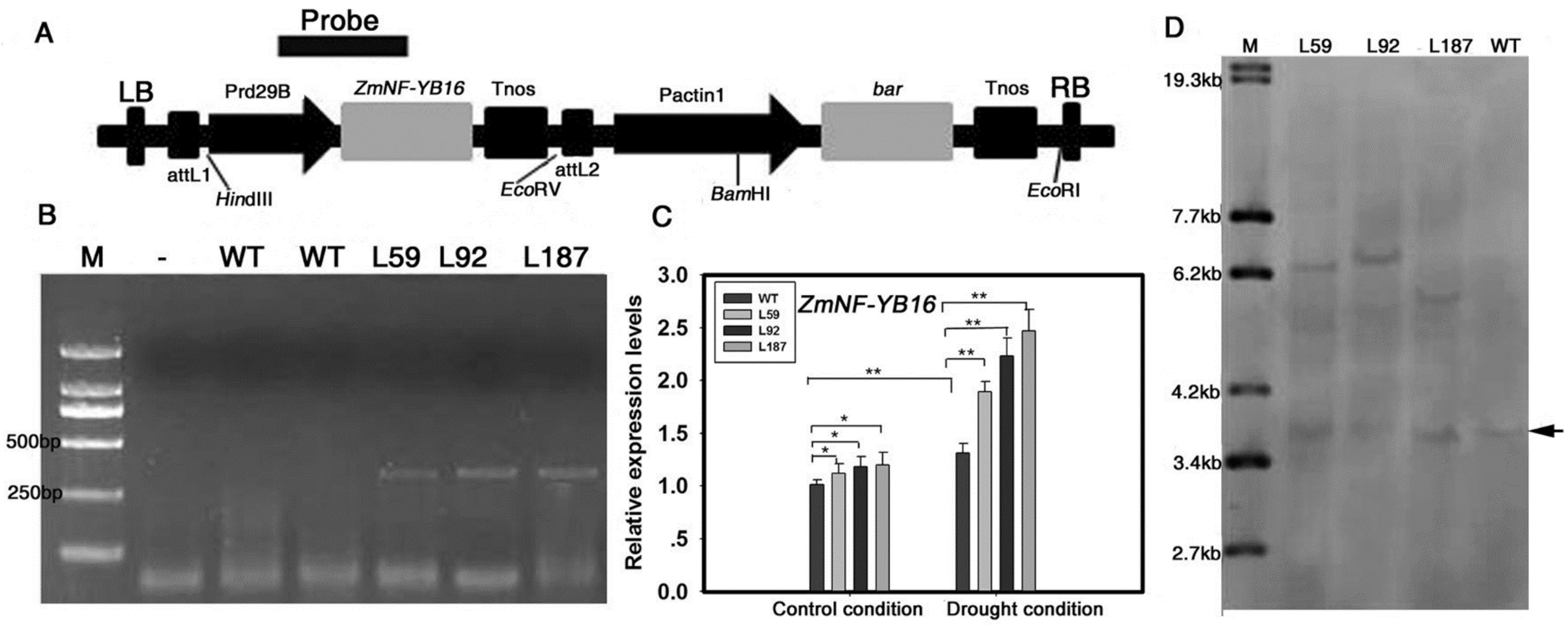

Figure S2 Schematic structure of the T-DNA region of plasmid pDAB101851- Prd29B::*ZmNF-YB16*

WT, non-transformed control; L59, L92, L187: *ZmNF-YB16* transgenic plants. (A) The T-DNA region has a ZmNF-YB16 driven by an *Atrd29B* promoter (from Arabidopsis, an abiotic stress-responsive promoter), and a bar gene (from Streptomyces hygroscopicus) driven by the *OsActin1* promoter. (B) PCR analysis of plants for the bar gene: M, DNA marker DL2000; (C) Analysis by qRT-PCR of the WT and *ZmNF-YB16* transgenic lines. Drought condition was described as soil water content below 20%. (D) Southern blot analysis indicating the presence of a *ZmNF-YB16* transgene in the genome of transgenic maize. DNA from the plants was digested with *Hin*dIII and *Nde*I. The fragment of endogenous gene was 3.8 kb (Figure S1), which was indicated by the arrows. M: λDNA/*Eco*T14 molecular weight marker. LB and RB mean the left and right T-DNA border sequences, respectively. AttL1 and attL2 represent the recombination sites of gateway system.

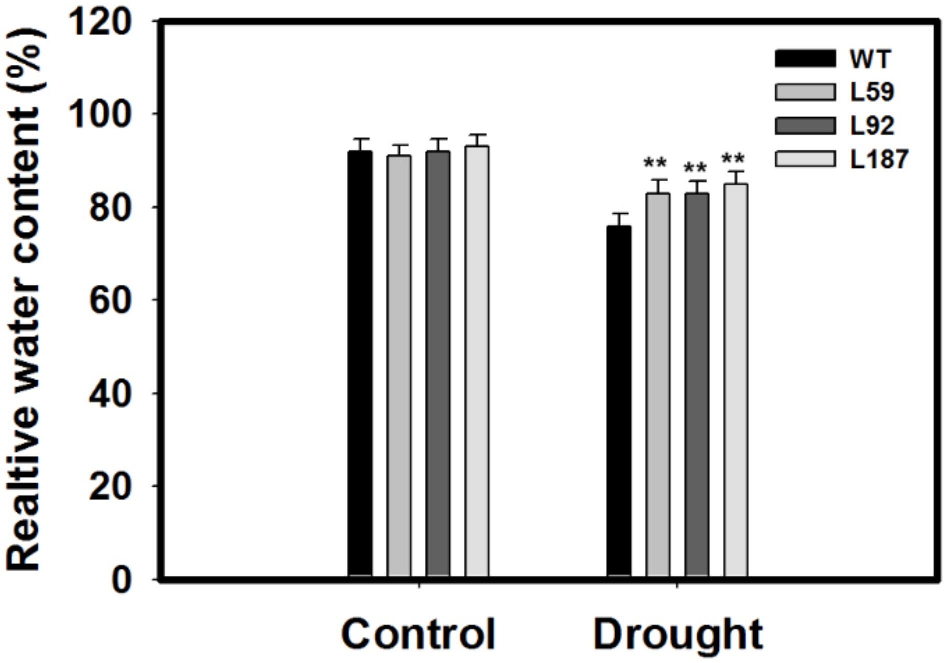

Figure S3 Relative water content of WT and transgenic plants under normal and drought conditions

Maize leaf at 5-leaf stage in flowering pots was used to measure the relative water content (RWC). Drought stress was imposed as relative soil moisture（RSM）kept with 45-50% for 10 days (when RSM was reduced 45-50%，1 d was recorded) (Avramova *et al.*, 2015), and control conditions was imposed as RSM kept with 85%-90%.

The asterisks indicate the significant difference between transgenic plants and WT in the same conditions using the *t-test*.*0.01 < P < 0.05 and **P < 0.01.

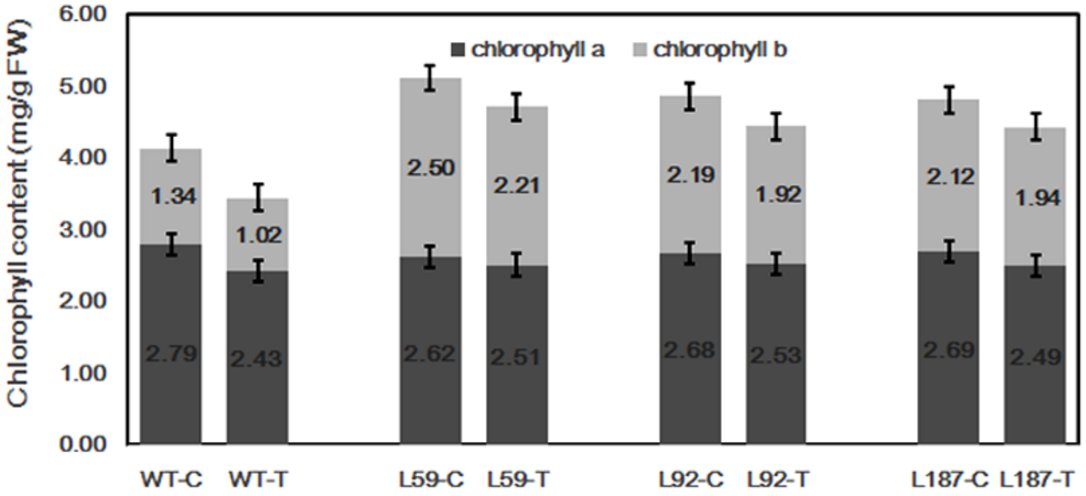

Figure S4 Chlorophy II content of WT and transgenic plants under normal and drought conditions

Note: WT-C: WT under control conditions; WT-T: WT under drought treatment conditions. FW: fresh weight. WT: wild type plants; L59, L92, L187: transgenic plants. Maize leaf at 10-leaf stage in flowering pots was used to measure the chlorophyll content. Drought stress was imposed as relative soil moisture（RSM）kept with 15% for 4 days (when RSM was reduced 15%，1 d was recorded), and control conditions was imposed as RSM kept with 85%-90%.
